# Supplementary material for: Spinal Manipulative Therapy for Acute Neck Pain: A Systematic Review and Meta-Analysis of Randomised Controlled Trials
Source: J Clin Med. 2021 Oct 28;10(21):5011. doi: 10.3390/jcm10215011 (PMC8584283; doi:10.3390/jcm10215011)
Supplement: Supplementary file 1 [file jcm-10-05011-s001.zip › jcm-1416769-supplementary.pdf]

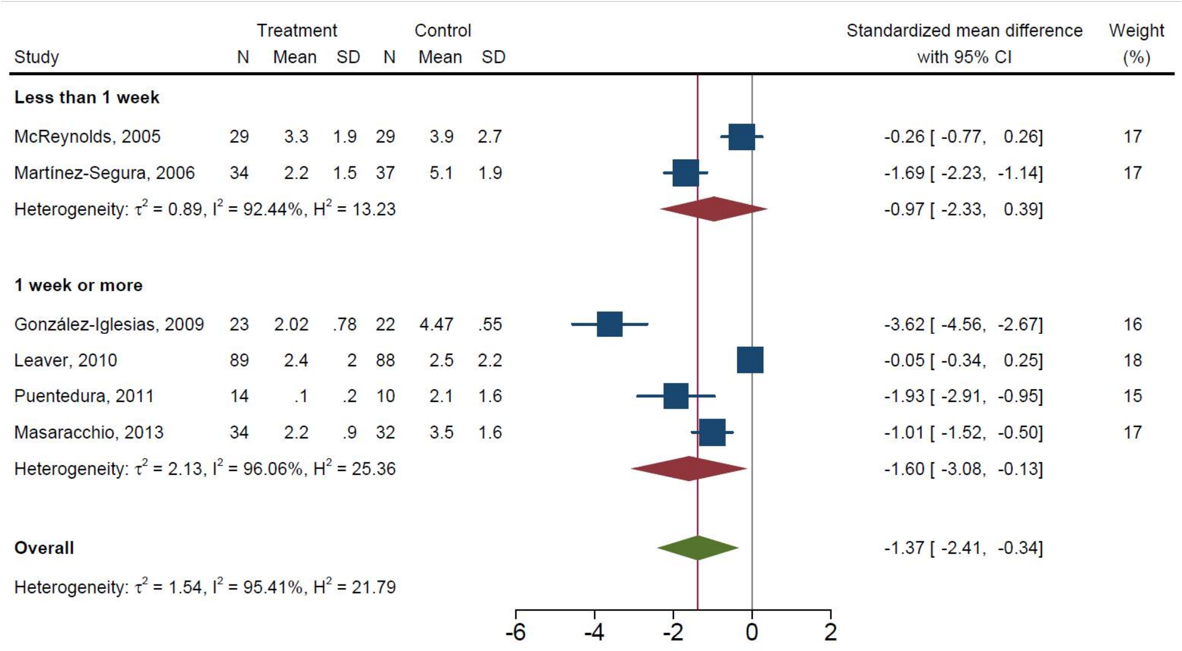

Figure S1. forest end by time.

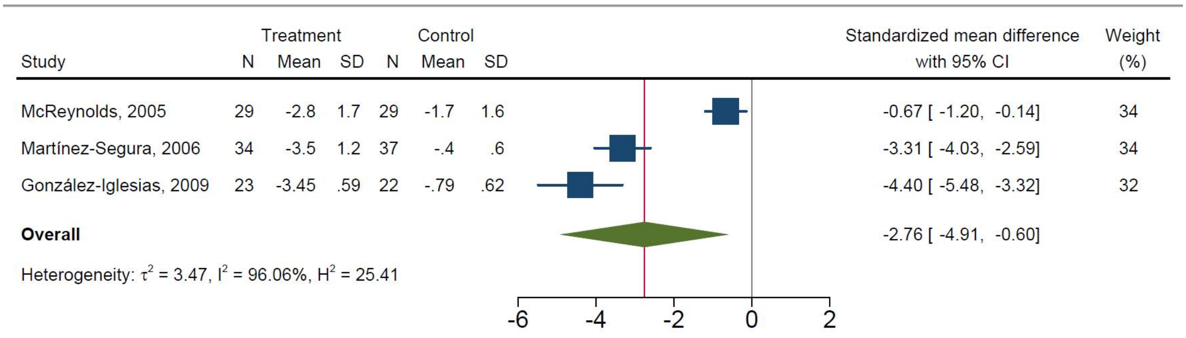

Figure S2. forest change overall.

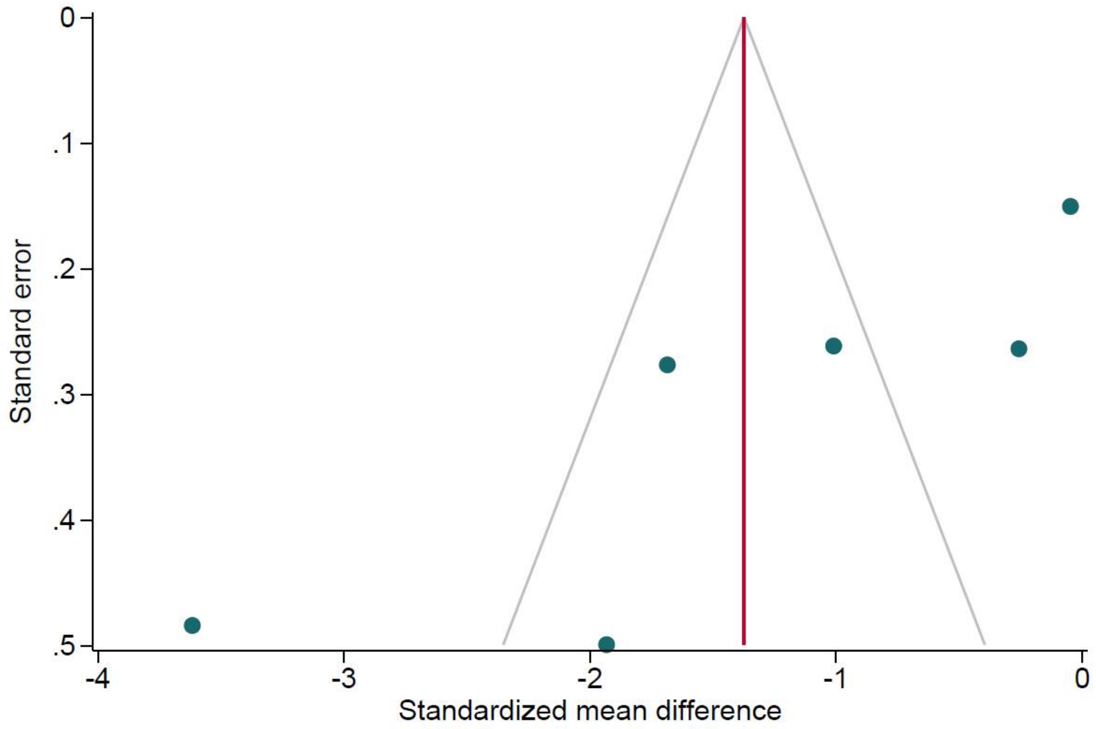

Figure S3. funnelplot overall.

Table S1. Rating of quality of the body of evidence across studies of short-term effects of manipulation therapy vs. control.

Item	Comment	Rating
Study design	The studies were all RCTs	High (RCTs)
Study limitations (risk of bias)	There were several threats to internal validity in the studies.<br>Lack of allocation concealment in 2 of 6 studies, and lack of blinding of participants and personnel<br>Dropouts: difference with regard to whether ITT was used<br>Only 3 of 6 studies reported change; possible bias related to selective reporting<br>Heterogeneity in interventions and control arms across studies<br>Heterogeneity in time for first or only outcome assessment: 1 h to 1 day in two studies; 1-3 week in other studies. Other periods not similar either (up to 6 months)<br>The studies used numeric rating scales (n=4) or visual analog scales (n=2), which may have different properties	-2
Indirectness of evidence	Limited scope: Short-term effect on pain. Might be other important outcomes.	0
Inconsistency of results	Considerable heterogeneity in the effects, as assessed by I^2 and a statistical test for heterogeneity. The effect varied by choice of rating scale, but not by other study characteristics, as indicated in exploratory meta-regression analyses.	-1
Imprecision	Most studies were small (24-71 patients), except for one with n=182. In total, n=441 participants were evaluated. The small size of the studies, lead to wide confidence intervals.	-1
Publication bias likely	Altogether few studies were identified. It is possible that studies were	-1

	unpublished or published in places that could not be identified (funnel plot, test of asymmetry).	
Dose–response	N/A	0
Plausible confounding	RCTs, therefore N/A	0
Magnitude of effect	As classified by the standardized response mean, the effect was large (SRM >1.3), although there is a risk of bias	0

The overall level of the body of evidence was rated as very low [⊕○○○], i.e. we have very little confidence in the effect estimate, and the true effect is likely to be substantially different from the estimate of effect (Balshem 2011).

Reference

1. Balshem, H.; Helfand, M.; Schünemann, H.J.; et al. GRADE guidelines: 3. Rating the quality of evidence. *J. Clin. Epidemiol.* **2011**, *64*, 401–406.
